# 组蛋白甲基化酶SMYD3在肿瘤中的研究进展

**DOI:** 10.3779/j.issn.1009-3419.2014.09.09

**Published:** 2014-09-20

**Affiliations:** 1 300052 天津，天津医科大学总医院心胸外科 Department of Cardiothoracic Surgery, Tianjin General Hospital, Tianjin 300052, China; 2 天津市肺癌研究所 Tianjin Lung Cancer Research Institute, Tianjin 300052, China

近年来肿瘤发病率逐年增高，严重威胁到人们的健康及社会发展，因此受到人们的广泛关注。现代肿瘤学理论观点认为，肿瘤的生物活性，不仅与经典的遗传机制(DNA核苷酸序列的改变)相关，还与表观遗传学(epigenetics)机制密切相关。

表观遗传学是研究DNA序列未发生改变而基因表达发生改变的科学，即通过对核小体上DNA和组蛋白的结构修饰及其之后的染色质重塑、非编码RNA调节等，从而在局部或整体上调节基因表达。肿瘤分子生物学研究表明，表观遗传学的紊乱与基因的变异一起参与了肿瘤发生发展的整个过程，其中包括肿瘤细胞增殖与分化、细胞周期的调控、肿瘤侵袭和转移以及肿瘤细胞逃避宿主免疫监视等。基因的表观遗传修饰主要包括DNA甲基化和组蛋白修饰^[1]^。其中组蛋白修饰，即核小体核心组蛋白在相关酶的作用下，其尾部常常发生翻译后修饰，包括甲基化、磷酸化、乙酰化、泛素化、腺苷酸化和ADP核糖基化等。这些修饰可以影响组蛋白与DNA双链的亲和力而改变染色质的状态，使其疏松或者凝集，也可以影响转录因子与结构基因DNA的启动子序列的结合，对基因表达调控具有类似DNA遗传密码的作用，故被称为"组蛋白密码"^[2-4]^。组蛋白氨基末端的修饰具有多面性、复杂性和特异性。近几年由于更多的组蛋白甲基化抗体的发现，针对组蛋白甲基化的研究日益增多。

组蛋白的甲基化修饰是指发生在H3和H4组蛋白N端精氨酸或者赖氨酸残基不同位点上的不同程度的甲基化，由含有SET结构域的组蛋白甲基转移酶介导催化完成。组蛋白甲基化的功能主要体现在异染色质形成、基因印记、X染色体失活和转录调控方面。目前已经发现和证实有大概700多种含SET[Su(var)3-9, Enhancer-of-zeste, Trithorax]结构域的组蛋白甲基转移酶，其中人源的就有100多种，它们参与了异染色质形成、基因印记、X染色体失活、染色体重塑、免疫生物学效应和转录调控等多种生理功能^[5]^。组蛋白甲基化酶的异常与多种人类疾病如肝癌等肿瘤、风湿等炎性疾病、喉软骨软化病等特殊疾病的发生密切相关^[6]^。SMYD3(SET and MYND domain containing 3)是近年来发现的一种具有组蛋白甲基化功能的蛋白。它能够使染色体组蛋白(H3K4等)发生二甲基化或三甲基化，作为RNA多聚酶复合物的一个成员，在转录调控中具有重要作用。它能够影响下游癌基因、细胞周期调控基因、信号转导相关基因、细胞核激素受体、粘附相关基因等，能够抑制肿瘤细胞凋亡、促进细胞增殖及侵袭、扩散^[7]^。多项研究^[7-9]^证实SMYD3在肝癌、结肠癌、乳腺癌等癌细胞中高表达，而在相应正常组织中表达量较低甚至检测不到。基于SMYD3对于组蛋白甲基化水平的影响与肿瘤的预后存在明显关联，另外*SMYD3*基因沉默的实验观察到明显的肿瘤细胞生长抑制、凋亡增加。可见，SMYD3与恶性肿瘤的发生发展及预后有密切关系，它能够通过不同的信号通路调控癌基因等许多与肿瘤发生发展相关基因的表达，并增强细胞的增殖和迁移能力。抑制SMYD3的表达可以阻碍肿瘤细胞生长、迁移并诱导凋亡，改善肿瘤预后，因此SMYD3日益成为人们关注的焦点。

## 1 SMYD3的结构和功能

2004年，Hamamoto等^[7]^通过cDNA芯片和半定量RT-PCR技术，在人类肝细胞癌和结肠癌细胞中首次发现了组蛋白甲基转移酶SMYD3^[10]^。*SMYD3*基因位于人类染色体1q44，由16个外显子组成，DNA(GenBank登录号：NM_64754)由1, 477个碱基对组成，其外显子可编码428个氨基酸的蛋白质。*SMYD3*基因所编码的蛋白为两叶结构，折叠成5个不同的结构域：SET domain、MYND、SET-Ⅰ、post-SET、C-terminal domain (CTD)。其中重要的两个功能结构域为：SET domain和MYND结构域。SET domain由两部分组成：S序列(S-sequence)与核心SET结构域(core SET domain)，两部分被MYND和SET-Ⅰ分开。然而，尽管在初级序列两部分是分隔的，但是在空间结构上两部分组合在一起折叠形成保守的SET结构域。SET结构域大约含有130个氨基酸，具有甲基化转移酶功能，可以特异性地使染色体组蛋白H3K4形成二甲基化或三甲基化，从而导致染色体空间结构成为松散开放的状态^[7]^。N端第1-28位氨基酸的MYND(myeloid translocation protein 8, Nervy, DEAF1)锌指功能结构域，能够特定地与相关基因启动子区域的基因序列5c-CCCTCC-3c或5c-GGAGGG-3c相结合，使SET结构域的甲基化功能发挥，影响基因转录；其表面带有大量的正电荷，通过一个富含脯氨酸的序列介导蛋白-蛋白相互作用及DNA与之结合，从而增强SMYD3的组蛋白甲基化转移酶活性。另外两个参与SMYD3活性位点形成的区域，一个是SET结构域后一个富含半胱氨酸的三级结构post-SET，能够结合锌离子；另一个是C端区域包含的一个三四重复氨基端序列(tetratrico-peptide repeat, TPR)结构。二者同SET结构域一起共同组成了一个深而窄的底物结合口袋，而第239位酪氨酸的羟基则是SMYD3发挥酶活性必要条件^[11]^。

SMYD3可以接受S-腺苷甲硫氨酸提供的甲基，使相关组蛋白H3赖氨酸4(H3K4)发生甲基化，造成染色质空间结构变化，从而改变转录复合物的紧凑程度和开放程度，从而对基因转录发挥调控作用。Sliva等^[12]^认为SMYD3 N端的SET-N区域可以抑制其甲基化转移酶的活性。SMYD3的甲基化作用可能是通过对其N端功能域的剪切去除或者与HSP90A发生相互作用而实现的。最近研究^[13, 14]^表明SMYD3同样可以作用于H4K20、H4K5。SMYD3也许可以通过其他作用位点促进肿瘤发生，新的作用位点有待进一步研究。

## 2 SMYD3与下游基因的关系

有研究通过cDNA芯片分析，发现SMYD3可以明显改变80个候选基因的表达，其中61个上调、19个下调。在这些相关的下游基因中，包括癌基因、抑癌基因、细胞周期调控基因、信号转导一系列基因等与肿瘤发生发展过程密切相关的基因^[15-17]^，如c-Met、雄激素受体(androgen receptor, AR) ^[18-20]^、基质金属蛋白酶(matrix metalloproteinases, MMP)-9、Myc^[21]^、CrkL、Wnt10b^[8]^、视网膜母细胞瘤蛋白结合锌指结构基因(retinoblastoma protein-interacting zinc finger gene, RIZ) ^[22]^、E2F转录因子1(E2F transcription factor 1, E2F1)、NK2同源序列8(Nkx2.8)、端粒酶反转录酶(human telomerase reverse transcriptase, hTERT) ^[15]^；控制细胞周期的基因如细胞周期蛋白G_1_、细胞周期素依赖激酶(cyclin-dependent kinase 2, CDK2)；控制信号传导的基因如信号传导与转录激活子(signal transducer and activator of transcription 1, STAT1)、丝裂原活化蛋白激酶(mitogen-activated protein kinase, MAP3K)、磷脂酰肌醇激酶(phosphoinositide 3-kinases, PIK3CB)；细胞核激素受体如孕激素受体亚型(progesterone receptor, PR-A) ^[23-25]^；转化生长因子(transforming growth factor, TGF)家族如肌生成抑制蛋白；血管内皮细胞生长因子受体1(vascular endothelial growth factor receptor 1, VEGFR1)；粘附相关基因如CD31、整合素α5。相关研究^[26]^表明，SMYD3可通过激活细胞外信号调节激酶/丝裂原活化蛋白激酶(extracellular signal regulated kinase/mitogen activated protein kinase, ERK/MAPK)信号转导通路和上调细胞周期相关基因细胞周期素D1(CyclinD1)和细胞周期素依赖激酶(cyclin-dependent kinase 4, CDK4)的水平提高肿瘤细胞的增殖能力。此外，*c-myc*基因是作为一个十分重要的原癌基因，在多种肿瘤组织中表达明显增加，c-myc激活能够促进细胞增殖，抑制凋亡。SMYD3通过直接或间接途径激活c-myc，从而发挥促进成瘤作用^[21]^。与SMYD3相关的另一个抑制癌基因*RIZ1*，它具有H3-K9组蛋白甲基转移酶活性，通过这一甲基化作用抑制相关基因的转录表达。在对包括肝癌、乳腺癌、结直肠癌等多种癌组织的检测中发现RIZ1低表达或者缺乏活性，研究表明这种情况可能与其启动子区域过甲基化有关。一个有趣的现象是在肝癌细胞中抑制SMYD3表达可能使过甲基化的RIZ1启动子区域去甲基化，进而RIZ1转录活性、甲基化活性都得以恢复，相反地，在RIZ1活性正常的细胞中转染SMYD3能够观察到RIZ1过甲基化的结果^[22]^。除此之外，VEGFR1作为SMYD3的作用靶点，也被相关研究^[17]^证实。新研究^[8]^发现，在乳腺癌的发生中SMYD3可能通过激活原癌基因WNT10B而发挥其致癌作用。同样在乳腺癌细胞中，SMYD3作为雌激素受体介导的转录辅助激活因子，使位于目标基因启动子区域的雌激素反应元件的H3K4甲基化。SMYD3表达下调可使*H3K4*甲基化减少，雌激素受体目标基因表达受到抑制^[8, 23, 27]^。MMP家族成员使细胞外基质(extracellular matrix, ECM)成分减少，同时激活生长因子。SMYD3表达沉默使*MMP-9*基因的启动子*H3K4*甲基化选择性减少，从而该基因表达减少。SMYD3过度表达导致MMP-9在白细胞转化、纤维肉瘤细胞、乳腺癌等表达。过氧化物酶15-LOX-1表达下调与许多恶性肿瘤的发病机理相关，这些肿瘤包括：霍奇金淋巴瘤(Hodgkin lymphoma, HL)、前列腺癌^[28]^、结直肠癌。SMYD3过度表达可诱导15-LOX-1启动子激活^[29]^。通过siRNA抑制*SMYD3*基因表达可以减少结肠癌细胞HCT116和霍奇金淋巴瘤细胞L1236的染色体*H3K4*三甲基化，并且减弱转录因子c-myc和SP1与*hTERT*基因启动子结合。由此可以看出，SMYD3在机体内发挥其活跃生物学功能，其更多的作用靶点、确切的作用机制尚待进一步阐明。

## 3 SMYD3与细胞生物学活性

细胞周期主要分4个时期：G_1_期(DNA合成前期)、S期(DNA合成期)、G_2_期(DNA合成后期)和M期(有丝分裂期)。细胞增殖调控包括两部分：环境中控制细胞增殖的因素及细胞内与细胞增殖有关的基因及其产物相互作用，即生长因子/生长因子受体如c-Met、细胞周期有关基因及其产物如CyclinD1、周期蛋白cyclinA、周期蛋白激酶CDK1等方面调控。染色体组蛋白修饰状态的改变可以影响细胞周期进程。Liu等^[18, 29]^研究认为SMYD3可以加快S期，促进细胞从S期向G_2_期过渡。但Zhao等^[30]^发现雷公藤甲素可以使SMYD3降低，引起骨髓瘤U266细胞G_2_/M期停滞。这可能与SMYD3的高度表达导致CDK2(cyclin dependent kinase 2)和DNA拓扑异构酶Ⅱβ等表达上调有关^[7]^，另外，hTERT编码端粒酶复合物的催化亚基，这是细胞永生化的先决条件。在*hTERT*基因启动子区域有5个SMYD3结合位点。SMYD3直接与端粒酶(hTERT)编码基因的启动子区域结合位点结合，上调hTERT mRNA表达，并增加端粒酶活性，直接介导细胞永生化，发挥促瘤效应^[15]^。

## 4 SMYD3与肿瘤之间的关系

国内外学者研究表明，SMYD3与肿瘤的发生发展关系密切，能够影响肿瘤细胞的增殖、凋亡、迁移及粘附等生物学行为。SMYD3与许多肿瘤相关如：结直肠癌、肝细胞癌^[7]^、胰腺癌、前列腺癌^[31, 32]^、多发性骨髓瘤、肺癌、乳腺癌、脑瘤^[33]^、食管鳞癌^[34]^等。有研究^[22]^发现，SMYD3在大多数肝细胞癌异常高表达，而在正常组织中不表达或表达很弱。采用siRNA技术使SMYD3表达沉默后，肝癌细胞凋亡有所增加，细胞的增殖也受到一定程度的抑制。Guo等^[9]^发现丙型肝炎病毒(hepatitis C virus, HCV)可以通过调节SMYD3和组蛋白甲基化使抑癌基因*RASSF1A*(ras association domain family 1A)启动子的甲基化水平上调。Zeng等^[35]^发现miR-124可抑制SMYD3表达，HCVc可以通过DNMT1使miR-124表达水平减低，SMYD3表达增高，可以促进胆管癌细胞转移和侵袭。Yang等^[36]^研究发现在乙型肝炎病毒(hepatitis B virus, HBV)(+)的细胞或组织中，SMYD3与HBX呈正相关；随着HBV沉默，SMYD3表达减少。Josse等^[37]^通过比较在PHH和HepaRG细胞中经黄曲霉毒素诱导的基因表达改变，发现在肝细胞中许多基因发生改变，特别是与p53和SMYD3相关的基因发生改变。Hamamoto等^[7]^发现SMYD3在结肠癌中表达的研究也提示明显增高，抑制其表达后结肠癌细胞生长和迁移明显受抑，凋亡增加。在乳腺癌的相关研究发现在大多数乳腺癌组织中SMYD3表达较正常组织明显增高，而利用siRNA技术抑制SMYD3的表达后可以改变乳腺癌细胞的细胞行为学特征，癌细胞出现生长受抑、凋亡增加等^[38]^。Luo等^[39]^发现SMYD3可作用于RhoA/MRTF-A(myocardin-related transcription factor-A)信号通路，SMYD3过度表达可促进MRTF-A介导上调肌球蛋白调节轻链9(MYL9)及MCF-7乳腺癌细胞转移。当内源性MRTF-A和SMYD3经特定的siRNAs抑制时，可观察到与之相反的结果。Wang等^[40]^研究发现下调*SMYD3*基因的表达后，宫颈癌细胞HeLa的增殖、迁移能力明显受限、集落形成减少、同时细胞凋亡有所增加。Liu等^[18]^发现SMYD3可促进前列腺癌发生，并且通过表观遗传学上调雄激素受体(androgen receptor, AR)表达。SMYD3缺失使SMYD3和/或AR的目标基因*Bcl-xL*、*MMP-9*、*hTERT*等表达下调有助于诱导细胞凋亡。另外，Nakamura等^[41]^通过实验研究发现，SMYD3在高转移性的胰腺癌中较正常组织表达有所升高，但作用机制尚需探讨。此外有研究称：在稳定转染人*SMYD3*基因后，小鼠成纤维细胞NIH3T3的生长和集落形成明显加快，细胞周期S期缩短，迁移和粘附能力增加，细胞的耐药性增强^[42]^。另外，*SMYD3*基因启动子区域的序列重复多态性是否和组蛋白甲基化状态、进而和肿瘤发生之间存在密切关系，目前存在争议。一项基于前列腺癌的研究认为：组蛋白H3的甲基化状态和前列腺癌患者的生存率以及肿瘤的生物学行为明显相关，并且这种甲基化状态的改变可能出现在染色质的分子结构变化之前，可能是肿瘤生物学行为的基础^[43]^。在结直肠癌、乳腺癌、肝癌细胞的相关研究表明*SMYD3*基因启动子区域的序列重复多态性是肿瘤发生的一个危险因素。Bernd等^[44]^发现，在家族聚集性乳腺癌患者中，*SMYD3*基因启动子去的序列重复多态性发生的概率更高。Mazur等^[45]^运用蛋白质芯片技术证实MAP3K2激酶是SMYD3的一个作用位点。SMYD3可使MAP3K2甲基化，激活MAP激酶信号通路，经Ras信号通路促瘤形成。运用胰腺导管癌和肺腺癌小鼠模型研究发现，降低SMYD3的催化活性可以抑制Ras引起的肿瘤发展。在肿瘤细胞系中，SMYD3介导MAP3K2的260赖氨酸甲基化，从而加强Ras/Raf/MEK/ERK信号通路活性。SMYD3缺失可以与MEK抑制剂协调作用，共同抑制Ras导致的肿瘤发展。作为MAP激酶通路的关键的负调节因子，PP2A磷酸酶复合物可以与MAP3K2结合，而甲基化阻断了它们的相互作用。研究结果证实赖氨酸甲基转移酶在细胞质激酶信号通路的新作用，并发现SMYD3在调节Ras致瘤信号通路中具有重要作用。

## 5 未来研究空间

综上所述，相关癌组织或癌细胞中高表达的*SMYD3*基因是研究肿瘤增殖、侵袭，以及对下游基因作用的研究焦点。目前的研究倾向于认为SMYD3可能在肿瘤形成过程中起重要作用，大量和SMYD3存在密切联系、又在肿瘤发生发展中起重要作用的蛋白或核酸分子被相继发现，但是SMYD3与下游因子的作用机制还没有得到确切的证据。因此，更广泛地研究SMYD3在肿瘤中的表达和作用，以及更深入地探讨SMYD3的作用机制是科学工作者进一步努力的方向。
